# From learners to healers: The essential link between student‐centred education and patient‐centred care

**DOI:** 10.1111/medu.70096

**Published:** 2025-11-29

**Authors:** Ligia Maria Cayres Ribeiro, Marco A. de Carvalho Filho

**Affiliations:** ^1^ Wenckebach Institute (WIOO), Lifelong Learning, Education, Assessment and Research Network (LEARN) University of Groningen, University Medical Center Groningen Groningen The Netherlands

## Abstract

From learners to healers: In this commentary, the authors discuss how shifting the culture of medical education from teacher to student‐centered is needed to fostering patient‐centered care.

In this issue, Huang et al.^1^ present a realist synthesis on how narrative medicine influences medical students' readiness for holistic care. Their findings suggest that storytelling, whether students' own or their patients', can foster empathy, emotional resonance, and deeper patient connection. However, some students engaging with narrative medicine reported unintended consequences, such as emotional avoidance and disengagement. In certain cases, they perceived the method as inauthentic. In others, they resisted adopting the patient's perspective. We believe these outcomes are not solely due to limitations in the instructional method itself but are deeply rooted in the broader culture of medical education, which often struggles to reconcile its pedagogical ideals with its structural realities.

The movement for patient or person‐centred care aims to deliver health care that is evidence‐based, clinically appropriate, and meaningful to the individual receiving it.^2^, ^3^ It requires recognising personal values and preferences, respecting cultural diversity, avoiding stereotypes, and promoting shared decision‐making.^2^ The concept first emerged in the 1950s and gained momentum in the 1990s, largely propelled by the patients' rights movement.^3^ Initially, patient‐centred care was viewed as a challenge to the dominant biomedical model and was even considered offensive by some practitioners who believed that patients had always been central to care.^3^ Others misunderstood it as relinquishing clinical authority to patients who might lack the necessary knowledge to make informed decisions.^3^ Despite these early tensions, the model gained legitimacy through growing evidence of its benefits, including improved patient satisfaction, better health outcomes, and enhanced therapeutic relationships.^2^ Today, patient‐centred care is widely accepted as a foundational principle of modern medicine.

Medical education has responded to the paradigm shift toward patient‐centred care by embedding its principles into competency frameworks and developing instructional strategies, such as reflection and narrative medicine, to cultivate empathy and holistic thinking. However, despite these efforts, the broader educational culture often sends conflicting messages that undermine these ideals. Several structural and cultural features of medical education mirror the very issues patient‐centred care seeks to address:

## 1 HIERARCHICAL RELATIONSHIPS

As the doctor–patient relationship has been traditionally hierarchical, so has the teacher–student. In clinical care, physicians held authority over patients, making decisions on their behalf. Similarly, in medical education, teachers often occupy a dominant role, with students expected to comply with the curriculum.^4^ When learners are treated as consumers rather than collaborators, it mirrors the paternalistic model of care that patient‐centred approaches seek to dismantle.

Physicians held authority over patients, making decisions on their behalf. Similarly, in medical education, teachers often occupy a dominant role.

## 2 STANDARDISATION

In both education and health care, standardisation aims to ensure consistency, safety, and quality. However, rigid adherence to standardised curricula in medical education can mirror the pitfalls of protocol‐driven care in clinical settings. Just as standardised treatment plans may overlook individual patient needs, standardised curricula may fail to accommodate diverse learning needs, backgrounds, and aspirations. This one‐size‐fits‐all approach can stifle motivation from patients and students.

Standardized curricula in medical education can mirror the pitfalls of protocol‐driven care in clinical settings.

## 3 FRAGMENTATION

Short clinical rotations and fragmented care both hinder the development of trust and continuity. In clinical settings, patients often see multiple providers, making it difficult to build therapeutic relationships. Similarly, students rotate through departments and supervisors rapidly, limiting opportunities for mentorship and meaningful feedback. These transient interactions can feel transactional rather than relational. Building trust requires time and continuity.

Short clinical rotations and fragmented care both hinder the development of trust and continuity.

## 4 DEPERSONALIZATION

Depersonalization is a pervasive issue in both medical education and clinical care. Patients are often referred to by their condition (‘the renal failure in room 12’), and students by their role (‘the resident’ or ‘the third‐year student’), rather than by name. This language reflects and reinforces a culture of objectification and massification. It erodes the recognition of individual identity, which is central to both healing and learning. When educators and clinicians fail to acknowledge the person behind the role, they risk fostering environments that are emotionally disengaged.

When educators and clinicians fail to acknowledge the person behind the role, they risk fostering environments that are emotionally disengaged.

## 5 SCEPTICISM

In both education and health care, there is often scepticism about individuals' capacity to participate meaningfully in shaping their own journey. Some clinicians doubt patients' ability to make informed decisions, just as some educators question students' readiness to cocreate their learning. Just as patients are often expected to follow treatment plans without adequate explanation, students are frequently asked to master topics that seem disconnected from clinical practice simply because they are deemed important. In both cases, this approach undermines autonomy and discourages meaningful engagement.

As patients are expected to follow treatment plans without explanation, students are asked to master topics simply because they are deemed important.

Hierarchical relationships, standardisation, fragmentation, depersonalization, and scepticism not only limit immediate participation but also shape future behaviour. When students are not trusted or empowered during their education, they may internalise these dynamics and replicate them in their relationship with patients. A teacher‐centred approach hinders the development of empathy, autonomy, and relational skills, making patient‐centred care feel artificial or disconnected from students' lived experiences. This disconnect can lead to emotional disengagement, which carries over into their professional behaviour and perpetuates the very hierarchies and depersonalization that patient‐centred care seeks to dismantle.

Rebalancing the doctor–patient relationship benefits from rebalancing the teacher–student relationship. Embracing flexibility through electives, reflective practices, and adaptive learning is possible and can foster deeper engagement and better prepare students to deliver individualised care.^5^ Educational models that prioritise longitudinal relationships, such as mentorship programmes or longitudinal integrated clerkships, help cultivate the same depth of connection that patient‐centred care strives for. Simple yet powerful acts, like calling people by name, acknowledging their stories, and treating them as whole individuals, can restore relational depth. Co‐construction, a pedagogical model in which students engage in dialogue, reflection, and shared decision‐making rather than passively receiving knowledge, also offers a promising path forward.^4^, ^6^, ^7^

In 1996, Laine and Davidoff wondered, in their *Patient‐Centred Medicine, A Professional Evolution*,^3^ whether patient‐centred care would endure an often inhospitable health care system. Over the following decades, patient‐centred care not only survived but also became a defining principle of modern clinical practice, shaping policies, professional standards, and educational frameworks. Student‐centred education has the potential to follow a comparable trajectory and, in turn, reinforce the values of patient‐centred care.

## AUTHOR CONTRIBUTIONS

**Ligia Maria Cayres Ribeiro:** Conceptualization; writing—original draft; writing—review and editing. **Marco A. de Carvalho Filho:** Conceptualization; writing—review and editing.

## Data Availability

Data sharing not applicable to this article as no datasets were generated or analysed during the current study.
